# ROS and TGFβ: from pancreatic tumour growth to metastasis

**DOI:** 10.1186/s13046-021-01960-4

**Published:** 2021-05-03

**Authors:** Chao-Hui Chang, Siim Pauklin

**Affiliations:** Botnar Research Centre, Nuffield Department of Orthopaedics, Rheumatology and Musculoskeletal Sciences, University of Oxford, Windmill Road, OX3 7LD Oxford, UK

**Keywords:** TGFβ signalling pathway, Reactive Oxygen Species (ROS), Epithelial‐to‐mesenchymal transition, Pancreatic cancer, Metastasis, Cancer stem cells (CSCs)

## Abstract

Transforming growth factor β (TGFβ) signalling pathway switches between anti-tumorigenic function at early stages of cancer formation and pro-tumorigenic effects at later stages promoting cancer metastasis. A similar contrasting role has been uncovered for reactive oxygen species (ROS) in pancreatic tumorigenesis. Down-regulation of ROS favours premalignant tumour development, while increasing ROS level in pancreatic ductal adenocarcinoma (PDAC) enhances metastasis. Given the functional resemblance, we propose that ROS-mediated processes converge with the spatial and temporal activation of TGFβ signalling and thereby differentially impact early tumour growth versus metastatic dissemination. TGFβ signalling and ROS could extensively orchestrate cellular processes and this concerted function can be utilized by cancer cells to facilitate their malignancy. In this article, we revisit the interplay of canonical and non-canonical TGFβ signalling with ROS throughout pancreatic tumorigenesis and metastasis. We also discuss recent insight that helps to understand their conflicting effects on different stages of tumour development. These considerations open new strategies in cancer therapeutics.

## Background

Transforming growth factor β (TGFβ) is a cytokine with a prominent role in cell growth and differentiation in many tissues as well as inflammatory processes, autoimmunity and tumorigenesis [1]. In normal circumstances, a basal level of TGFβ signal is kept by local sources to maintain tissue homeostasis. Upon tissue injury, TGFβ is abundantly released by blood platelets and various stromal components for tissue repair, wound healing and for attenuating inflammation. TGFβ signalling plays a central role in tumorigenic processes depending on the timing and cell context. In early stages of tumorigenesis, the TGFβ signalling functions as an anti-tumorigenic signal while at later stages it exerts a pro-tumorigenic function by promoting epithelial to mesenchymal transition (EMT), cancer cell dissemination and metastasis [1]. Among the multiple mechanisms that mediate these contrasting effects is the interplay between canonical (Smad-mediated) and non-canonical (e.g. Kirsten rat sarcoma viral- extracellular signal-regulated kinase (KRAS-ERK), c-Jun N-terminal kinase/ p38 mitogen-activated protein kinase (JNK/p38), phosphatidylinositol-3-kinase- protein kinase B (PI3K-AKT), nuclear factor kappa B (NFκB)) signalling that are crucial for determining the differential effects on tumour suppression or tumour promotion.

 An intriguing determinant is the crosstalk between TGFβ signalling cascades and the partially reduced metabolites of oxygen molecules, knows as “reactive oxygen species” (ROS). ROS are free radicals that include hydroxyl ion, superoxide and hydrogen peroxide. They are produced in mitochondria, peroxisomes and endoplasmic reticulum, as well as by enzymatic reactions, such as cyclooxygenases, NADPH oxidases (NOXs), xanthine oxidases and lipoxygenases, and through iron-catalysed Fenton reaction. Moreover, ultraviolet rays, chemotherapy and radiotherapy can also stimulate ROS production. ROS also serves as secondary messengers mediating cellular functions and tumorigenesis [2]. Low level of ROS has important functions on cell fate and cellular responses impacting proliferation, differentiation and cell death [3], similarly to TGFβ signalling. However, when the level of ROS exceeds the antioxidant defence mechanisms, the imbalance leads to oxidative stress that causes direct or indirect damage of nucleic acids, proteins, and lipids [4]. Oxidative stress is common in cancers and accompanies high metabolic rates and genetic mutations in tumour cells, or hypoxic tumour microenvironment.

Both TGFβ signalling and ROS can exert anti-tumour effects by inducing apoptosis, senescence and cell cycle arrest, and pro-tumour effects by contributing to cancer cell movement, dissemination during metastasis, cellular proliferation, and survival. It is increasingly clear that the anti-tumorigenic versus pro-tumorigenic effects of ROS and TGFβ signalling have threshold levels and a cell specific effect during cancer development since cells from normal tissue respond differently from neoplastic cells, which are in turn different from metastatic cancer cells. TGFβ has been shown to modulate ROS production and thereby induce oxidative stress or redox imbalance in cancers, while ROS can in turn activate TGFβ. Research from recent years has provided intriguing insight to ROS-dependent pancreatic ductal adenocarcinoma (PDAC) formation helping to rationalize these conflicting reports of pro- and anti-tumour effects of antioxidant treatment [5, 6].

In this article we revisit the interplay between TGFβ signalling and ROS particularly in pancreatic tissue and throughout tumorigenesis in progression and metastasis. We discuss recent insight that helps to understand the contrasting effects of TGFβ signalling and ROS on early stages of tumour growth versus the metastatic processes, and how these discoveries impact therapeutic strategies for targeting ROS and TGFβ signalling in PDAC.

## The crosstalk between TGFβ signalling and ROS

### ROS activity regulated by TGFβ

Mitochondria provide a major source of ROS in cells. TGFβ has been shown to increase mitochondrial ROS production in various cell types via different mechanisms. TGFβ can directly induce ROS production in mitochondria via downregulation of complex IV activity leading to lung epithelial cell cycle arrest and apoptosis [7]. Mitochondrial complex III activity is required for TGF-β-mediated ROS generation and fibrogenetic gene expression, such as α-smooth muscle Actin (α-SMA) and connective tissue growth factor (CTGF), in normal human lung fibroblasts [8]. In mammary epithelial cells, the stimulation of ROS by TGFβ is interfered by exogenous expression of thioredoxin which then abrogates TGFβ-mediate EMT [9].

Apart from direct regulation on mitochondrial ROS production, numbers of NADH oxidase (NOX) and antioxidant enzymes have been reported as TGFβ-Smads-dependent. NOX4 induced by TGFβ has been studied the most with accumulating evidences in TGFβ-induced tissue fibrosis, though less been reported in cancers. For example, NOX4 is Smad3-regulated in breast cancers [10], and it provides ROS sources for the EMT phenotype switch in pancreatic cancers [11]. Besides, TGFβ can also increase ROS levels by repressing several antioxidant enzymes including glutaredoxin, catalase, superoxide dismutase, glutathione peroxidase, and by decreasing the concentration of glutathione (GSH), the most abundant intracellular free thiol and an important antioxidant. *De novo* GSH synthesis involves two-step catalysation by glutamate cysteine ligase (GCL) and GSH synthase (GS). TGFβ can inhibit the expression of the rate limitation enzyme, GCL via regulating the binding of c-Jun (also known as activator protein 1, AP-1)/Fos-related antigen 1 (Fra-1) complex, Smad3 and ATF3 transcription factors to GCL promoter (Reviewed in [12]). It hence suppresses GCL expression and GSH concentration and leads to an increasing ROS production in cells. Another TGFβ/Smad-dependent antioxidant, TIGAR (TP53 induced glycolysis regulatory phosphatase), has been described suppressing ROS level in glioma cells [13], lung fibrosis [14] and more recently in pancreatic cancer [6]. Of note, sustained ROS, in turn, negatively feedbacks on TGFβ pathway molecules.

### TGFβ ligand activation and ROS

Unlike most of the growth factors, TGFβ is deposited as a part of a latent complex (L-TGFβ) into the extracellular matrix (ECM). The TGFβ ligand acts as a molecular sensor in spatial and temporal way after responding to environmental perturbations. It is known that integrins, low pH, thrombospondin-1 (TSP1) and ROS are able to activate TGFβ (Reviewed in [12]). ROS can activate latent TGFβ through direct oxidation of latency-associated peptide (LAP) and indirectly through activation of MMP-2 (matrix metalloproteinase-2) and MMP-9 which in turn cleave LAP to release active TGFβ. The L-TGFβ1 contains a redox switch centered at methionine 253, allowing the ligand to act uniquely as an extracellular sensor of oxidative stress in tissues [15].

### Modulation of TGFβ signalling by ROS

In addition to activating TGFβ from its latent form, ROS can stimulate the expression and secretion of TGFβ, as well as act as mediator in the canonical and non-canonical pathways. Numerous studies showed that ROS can upregulate TGFβ gene expression in various types of cells. In cultured A549 human epithelial cells, ROS increases TGFβ production via NFκB and activator protein 1 (AP-1) mediated transcriptional regulations [16]. The expression of TGFβ2 and TGFβ receptor II were regulated by N-acetylcysteine (NAC), a ROS scavenger, in articular chondrocytes [17]. Also, ROS mediated TGFβ-regulated tissue inhibitor of metalloproteinase 3 (TIMP3) gene expression in chondrocyte through Smad2 but not ERK signalling [18]. ROS can induce TGFβ expression during EMT induction, suggesting the possibility of a TGFβ/ROS/TGFβ feedback loop in human keratinocytes [19]. Oxidative stress induced the conversion of endothelial cells into myofibroblasts through inducing mRNA and protein expression of both TGFβ1 and TGFβ2 [20]. ROS can induce TGFβ expression via p38/JNK/ERK and NFκB pathways in human hepatocellular carcinoma [21]. ROS mediates the TGFβ-dependent fibrogenic effects via Smad, PI3K, mitogen-activated protein kinase (MAPK), and Ras homolog family member A/Rho-associated protein kinase (RhoA/ROCK) pathways (reviewed in [22]) that can restrict access of therapeutics as a physical barrier. By oxidising active sites in phosphatases, ROS can restrain dephosphorylation on TGFβ-induced MAPK and facilitate the TGFβ/MAPK signalling (Reviewed in [12]). NOX4-derived ROS has been reported responsible for TGFβ induced pancreatic cancer cell chemotaxis via NOX4/ROS/p38 MAPK cascade [23]. Although many studies have demonstrated ROS induce TGFβ expression, how and which pathways involved in pancreatic cancers are not fully understood.

### TGFβ and ROS share downstream mediators

**Fig. 1:**
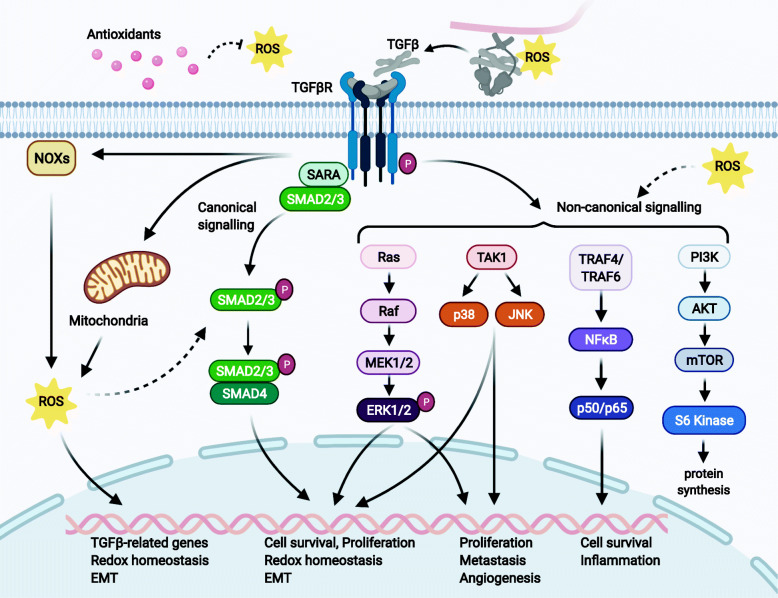
The crosstalk between TGFβ ligand activation, ROS levels and the canonical and non-canonical TGFβ pathways. ROS can activate latent TGFβ ligand in extracellular matrix but also stimulate the expression and secretion of TGFβ, and mediate the effects of TGFβ by modulating the activities of both canonical Smads pathway and non-canonical pathways: Ras-Raf-MEK-ERK, TAK1-JNK/p38, TRAFs-NFκB, and PI3K-AKT. In turn, TGFβ can directly stimulate ROS production through mitochondria and Noxs. TGFβ can also increase ROS levels through the canonical Smad2/3/4 pathway and non-canonical MEK/ERK pathway by suppressing the expression of several antioxidant enzymes or stimulating the expression of NOXs genes. Direct (solid arrows) or indirect (dash arrows) activation, and indirection inhibition (dash inhibitor) are shown

The canonical TGFβ signalling pathway is mediated by phosphorylated Smad2/3 proteins that act as transcriptional regulators in complex with Smad4. However, TGFβ receptors can also activate non-canonical non-Smad mediated pathways including MAPKs, PI3K, NFκB and Ras (Fig. 1). In pancreatic acinar cells, TGFβ induces a delayed ERK activation with peak phosphorylation after several hours, implying an indirect mechanism that requires de novo protein synthesis [24]. However, TGFβ can also activate RAS-RAF-MEK-ERK signalling with ERK phosphorylation rapidly within 5–10 min of TGFβ stimulation, which is comparable to the time course of ERK activation by mitogenic factors such as epidermal growth factor (EGF) [25]. ERK then regulates target gene transcription through its downstream transcription factors in conjunction with SMADs to control EMT. Interestingly, ROS is also able to activate MAPK pathways (e.g. ERK, p38 and JNK) and mediates transcriptional activity of NFκB, indicating that factors downstream of ROS and TGFβ receptors are shared between pathways [26].

The regulatory loop between TGFβ and ROS exists in cancer cells. TGFβ regulates mitochondrial ROS production directly and via regulating antioxidative enzymes expression [12]. The increasement of ROS causes genetic instability that may contribute to cancer initiation. In turn, ROS co-mediates TGFβ downstream signalling molecules which inhibits tumour proliferation in the initial stage of tumorigenesis (e.g. Smads), and switch to promoting metastasis in advanced stage (e.g. MAPKs, RhoA/Rho and NFκB). Moreover, ROS activates latent TGFβ complex in ECM and thereby sustains TGFβ signalling in the microenvironment [15]. Together, the ROS-TGFβ interplay strongly contributes to tumorigenesis while exerting multiple roles depending on the stage of malignancy.

## ROS and TGFβ in benign tissue and precancerous lesions

### Benign cells in pancreatic tissue

Elevated ROS has foremostly an anti-proliferative effect in benign pancreatic tissue by activating the DNA damage response pathways that block cell proliferation or lead to apoptosis upon too extensive cellular damage [27]. In normal cells, ROS can activate Src family, small G proteins, such as RAS, and PI3K/Akt pathway by inactivating phosphatase and tensin homolog (PTEN). A small increase in ROS level prefers PI3K/Akt pathway activation, and further increase can trigger JNK and p38 MAPKs pathways to induce cell apoptosis [2]. ROS is reported to activate ERK, JNK and p38 MAPK in the isolated pancreatic acinar cells [28]. This is particularly important to protection pancreatic tissue from damages such as acute pancreatitis. On the other hand, TGFβ signal is anti-proliferative in benign and well-differentiated pancreatic cells by inducing cell cycle inhibitors. The intersection between TGFβ and the RAS-ERK pathway is of particular interest for the prevalence of both KRAS mutations and altered TGFβ signalling in pancreatic cancer. KRAS mutations and altered TGFβ signalling are observed in > 90 % of PDAC [29]. Oncogenic KRas is capable to initiate PDAC in murine model, which recapitulates the human disease [30, 31]. Ras-ERK pathway mediates not only PDAC initiation, but also tumour growth and maintenance [32]. Most studies of ERK have focused on growth, proliferation and regeneration as it is recognized as a major regulator of G1 and S phase transition. Interestingly, the inhibition of ERK phosphorylation alleviates TGFβ-induced SMAD2 phosphorylation and p21 upregulation in benign pancreas duct cells, while preventing suppression of the pro-growth signal cyclin-dependent kinase 2 (CDK2) and ablating TGFβ-induced EMT [33]. Similarly to TGFβ, constitutional activation of RAS increases ROS production via Rac Family Small GTPase 1 (Rac1)/NOX4 [4] and mitochondria [34] in pancreatic acinar cells.

### Pre‐cancerous lesions

During the earliest stages in pancreatic cancer development, pancreatic acinar cells undergo trans-differentiation into duct-like cells, a process called acinar-to-ductal metaplasia (ADM) which progress to pancreatic intraepithelial neoplasia (PanIN) lesions that ultimately lead to PDAC. A gradual increase in ROS production has been observed in regions of ADM and in different PanIN states with the formation of a dense stroma and hypoxic environment that triggers metabolic changes and improves cell survival [34]. The increase of ROS in PanINs is not merely correlative, since mutant KRAS-induced mitochondrial ROS plays a central role in inducing the formation of precancerous lesions in the pancreas via upregulating epidermal growth factor receptor (EGFR) signalling through NFκB, oxidative stress sensor protein kinase D1 (PKD1), and tumour protein P53 inducible nuclear protein 1 (Tp53INP1) [34, 35]. The response of neoplastic PanIN cells is different from normal cells in terms of TGFβ and ERK signalling, a shared mediator of ROS [33]. In these cells, a partial divergence between TGFβ and MEK/ERK is observed where pERK is required for upregulation of p21 and EMT, but not necessary for TGFβ-induced pSMAD2 phosphorylation or CDK2 repression [33]. Hence, neoplastic cells are beginning to show some of the mechanistic changes in TGFβ/ROS/ERK signalling that sets normal pancreatic cells apart from PDAC cells.

## Dynamic interplay between ROS and TGFβ regulates tumour growth and metastasis

In contrast to benign pancreatic cells, ROS is important for inducing PDAC cell proliferation and survival. The growth promoting effects on this cancer cell context are mediated by ERK1/2 induction [36], PKD1-NFκB pathway [34], PI3K/Akt-NFκB pathway and Rac1 [3]. Notably, all these factors also crosstalk to TGFβ signalling pathway or are direct mediators of the non-canonical TGFβ signalling pathways. In PDAC cells, ERK has no effect on TGFβ-induced upregulation of pSMAD2 and p21, suggesting the two pathways have completely separated with respect to the cell cycle. Furthermore, pERK acts as a tumour promoter by positively regulating CDK2 and EMT, independent of exogenous TGFβ. This indicates that during carcinogenesis pERK engagement changes from initial facilitating to later antagonising TGFβ-mediated cell cycle arrest, yet it remains critical for the pathological, EMT-inducing arm of TGFβ signalling [33].

Oxidative stress has been shown to initiate hypoxia dependent EMT in PDAC cells. Using mutant KRAS-driven PDAC mouse models, TIGAR deletion delayed the emergence of premalignant PanIN lesions while increasing ROS [6]. On the other hand, loss of TIGAR and the increasing production of mitochondrial ROS enhanced the metastatic capacity of the tumour cells. TIGAR-deficient PDAC cells enhanced ERK signalling which drove collagen-degrading activity and promoted migration and invasiveness. The cellular functions responded to antioxidant treatment collectively indicated that limited ROS supports the establishment of the primary pancreatic malignancy and distant metastasis, while elevated ROS promotes metastatic spread of PDAC.

Since TGFβ has similar contrasting roles in cancer progression, the phenotypic switch of cancer cells due to ROS could also be relevant in the context of TGFβ pathway activation and EMT. Indeed, contrary to normal cells, TGFβ has been shown to promote the progression and metastasis of advanced cancers. TGFβ-enhanced invasive capacity of pancreatic cancer cells cross-talks extensively with ROS signalling and is mediated by Rac1, NFκB, IL-6 and MMP-2 [37]. Furthermore, ROS signalling contributes to TGFβ-induced EMT-like phenotype by augmenting migration through increasing SNAIL and SLUG while decreasing E-cadherin in PDAC [6], an effect that is also controlled by MAPK-activated Ras responsive element binding protein 1 (RREB1) and Smad2/3 cooperation on Snail promoter [38]. The non-canonical TGFβ-MEK/ERK pathway mediates the acquisition of mesenchymal phenotypes while inhibition of MEK/ERK prevents TGFβ-induced EMT in PDAC. One additional consideration could be that TGFβ signalling gradually/partially loses its control on antioxidation while ROS level remains high. The imbalanced regulatory loop consequently could favour EMT phenotypic switch, potentially via MEK/ERK signalling. In line with this, TIGAR expression is SMAD dependent while SMAD4 deficiency is commonly observed in a later stage of pancreatic cancer.

**Fig. 2:**
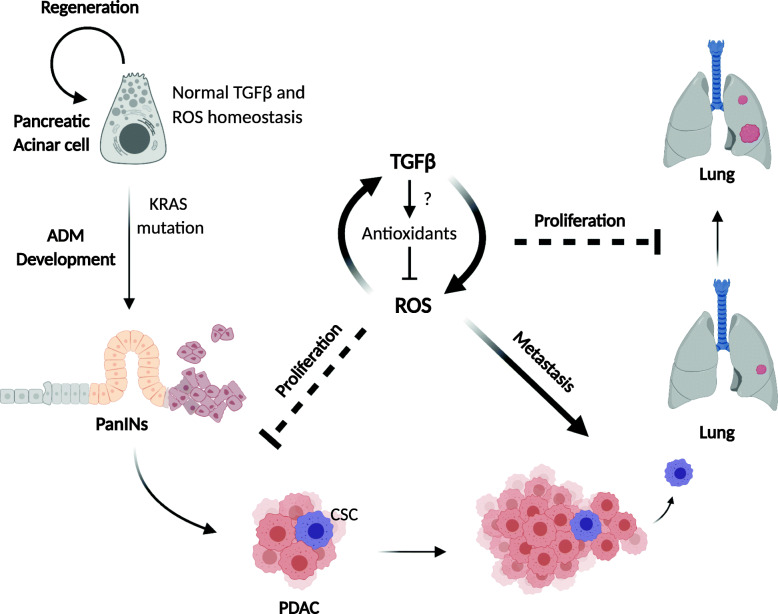
The proposed cooperation of ROS and TGFβ ligand activation in inducing EMT, invasion and metastasis. Pancreatic acinar cells in adult tissue show high plasticity to under go acinar-to‑ductal metaplasia (ADM), a reversible progress for pancreatic regeneration after injury such as pancreatitis. The progress becomes irreversible upon oncogenic KRAS mutation which leads to pancreatic intraepithelial neoplasia (PanIN) lesions and pancreatic ductal adenocarcinoma (PDAC). Antioxidant expression such as TIGAR is dynamically regulated during the development of pancreatic ductal adenocarcinoma, resulting in lower levels of ROS to promote tumour initiation in the premalignant condition and higher levels of ROS that enable metastatic progression. TGFβ signalling is expected to cooperate with ROS signalling to control EMT, invasion and metastasis in the cancer stem cell (CSC) subpopulation of cells that are particularly metastatic and able to switch their metabolic state due to developmentally plasticity. The PDAC progression from pancreatic acinar cell to metastasis in lung are shown in arrows. The positive and negative interplays between TGFβ, antioxidants and ROS, as well as their positive impacts to cancer progression are shown in arrows and dash inhibitors, respectively

Interestingly, ROS-dependent control of PDAC allows cells to switch between an epithelial/less invasive state and a mesenchymal/more invasive state [6], suggesting a regulation by reversible epigenetic mechanisms (Fig. 2). This phenotypic plasticity is observed in cancer stem cells (CSCs) that can metastatically disseminate to distant sites. Hence, cancer cells would be expected to express high levels of TIGAR and other antioxidant enzymes in favour of tumour growth [6]. Once dedifferentiating to CSCs that have elevated metastatic capacity, these cells could temporarily express low levels of TIGAR and produce elevated ROS and activate TGFβ with EMT. Upon reaching the secondary site, CSCs would switch back to higher TIGAR expression with the accompanying metabolic state with low ROS that would be more compatible with cell proliferation and tumour growth [6]. Collectively the interplay between ROS and TGFβ is likely to have an important role in tumorigenesis.

### Metabolic switching and ROS production in cancer

Oxidative phosphorylation in the mitochondria and glycolysis in the cytosol are two major metabolic routes that produce adenosine triphosphate (ATP) in mammalian cells. Normal cells in physiological conditions utilize the more energy efficient oxidative phosphorylation as the major pathway to generate ATP. In contrast, cancer cells utilize the glycolytic pathway for ATP production, even in the presence of oxygen. This switch to aerobic glycolysis in cancer cells is known as the Warburg effect. The glycolytic pathway also provides metabolic intermediates such as nucleic acids, proteins and lipids for cell growth and proliferation [39].

Normal stem cells have lower ROS levels and reduced oxidative damage due to elevated aerobic glycolysis in comparison to differentiated cells [40]. Metabolic transition from oxidative metabolism to glycolysis accompanies the epigenetic reprogramming of differentiated somatic cells to a pluripotent state [41]. Similarly, CSCs produce less ROS and have a lower energy metabolism rate compared with non-CSCs [42, 43]. This can be achieved by several processes that involve (i) increased glycolysis, (ii) upregulation of ROS scavengers, (iii) downregulation of ROS-producing enzymes, (iv) reduced mitochondrial mass, and (v) low oxygen consumption [44–46].

###  Cooperation of ROS, TGFβ and hypoxia inducible factors (HIFs) in CSCs

The normal physiological oxygen percentage in healthy tissue is approximately 7 % while in tumours it can range from physiological to severe (< 1 %) hypoxia [47]. Hypoxic condition is reported in various solid tumours among which PDAC is identified as the most hypoxic [48, 49]. In hypoxic conditions elevated ROS can activate hypoxia inducible factors (HIFs), heterodimers of HIF-1α, HIF-2α, or HIF-3α and HIF-β/aryl hydrocarbon receptor nuclear translocator (ARNT). While HIF-β is ubiquitously expressed in various cell types, HIF-α subunits are regulated by intracellular oxygen sensors known as prolyl hydroxylate enzymes and asparaginyl hydroxylase [50]. Interestingly, hypoxia-activated HIF proteins and TGFβ-activated SMAD2/3 proteins can cooperatively regulate gene expression including *PLOD2*, a key enzyme for proper deposition of collagen into the ECM [51].

It has become increasingly clear that a hypoxic microenvironment is beneficial for the maintenance of CSCs in virtually all tissues of the body by promoting the undifferentiated state of CSCs through inducing stem cell markers, increasing colony-forming capacity, invasiveness and resistance to therapeutics [52]. Low concentration of ROS can maintain the stemness of CSCs and induce tumorigenesis through HIF stabilization. Low-dose gemcitabine, an anti-cancer chemotherapy drug, can induce metabolic reprogramming toward aerobic glycolysis, promoting PDAC cell stem-like properties and chemoresistance. Mechanistically, gemcitabine-induced metabolic reprogramming and cancer stemness are regulated by ROS-mediated activation of the KRAS/AMP-activated protein kinase (AMPK) pathway [53]. Induction of mitochondrial dysfunction is an important mechanism by which KRAS signalling causes metabolic changes and ROS stress in cancer cells and promotes tumour development. Oncogenic activation of KRAS^G12V^ leads to mitochondrial dysfunction with decreased respiration, elevated glycolysis, and increased generation of ROS [54]. Furthermore, PI3K-AKT-mechanistic target of rapamycin (mTOR) signalling pathway can promote the synthesis of HIF-α, while inhibition of hydroxylase activity can prevent HIF-α degradation [55]. HIFs induce metabolic reprogramming from oxidative phosphorylation to anaerobic glycolysis as well as lactic acid fermentation, by activating lactate dehydrogenase A and phosphorylating the E1α subunit of pyruvate dehydrogenase. This helps to solve the energy requirement by providing more ATP for cancer cells and supports cell survival in the hypoxic condition by reducing cytotoxic ROS levels while also increasing resistance to chemo- and radiotherapy [56]. HIF-2α promotes the expression of multiple antioxidant enzymes and DNA damage repair enzymes, thereby reducing the intracellular ROS levels and limiting the accumulation of DNA damage [57]. HIF-1α is stabilized under severe hypoxia (1 %) while having only a little activity at 5 % O2 which correspond approximately to end-capillary oxygen conditions [58]. HIF-2α is stabilized more broadly, from severe hypoxia (< 1 % oxygen) to more physiologically relevant tension in tumours (2–5 % oxygen) [58, 59]. HIF-1α and HIF-2α are highly homologous and bind to similar hypoxic response have different biological functions due to different expression and binding to unique target genes. HIF-2α is mostly expressed in CSCs but not in non-CSCs in gliomas and it can induce Oct4, Glut1 and vascular endothelial growth factor (VEGF), thereby promoting CSCs in metabolism, proliferation, survival, and escape from immune surveillance [59]. On the other hand, HIF-1α was present in both CSC and non-CSC tumour subpopulations upon hypoxia. HIF factors regulate tumorigenic capacity and their expression is associated with higher cancer patient mortality [60]. HIFs also induce CSC cell-surface markers including CD133, CD44, and VEGF-A, and stem cell factors Oct4, Nanog, Klf4, Sox2 and c-Myc thus regulating CSC self-renewal [61].

### TGFβ and ROS contribute to PDAC fibrosis

PDAC is characterised by a high level of fibrotic reaction in tumour tissue, also known as desmoplasia. Stromal stiffness around tumour lesions accelerates tumour progression and associates with lower overall survival in PDAC [62]. Also, highly dense fibrotic stroma can cause burden in radiotherapy and impair drug delivery, leading to treatment failure [63]. Molecular studies indicate TGFβ signalling is a key player in the normal and pathological fibrosis in various tissues including pancreas [64, 65]. In pancreatic epithelial cells, altered TGFβ signalling due to SMAD4 mutation leads to high epithelial tension and increasing collagen I thickness through activation of JAK–STAT3 and integrin-focal adhesion kinase (FAK)/ROCK signalling [62]. Overexpression of TGFβ in transgenic mice shows similar pancreas morphology of chronic pancreatitis, including accumulation of ECM components and increasing number of pancreatic stellate cells (PSCs), a major promoter of pancreatic desmoplasia [66]. Inhibition of TGFβ, by contrast, protect mice from developing caerulein-induced pancreatic fibrosis [67]. Moreover, selective loss of TGFβ signalling in PSCs decreases the synthesis of ECM proteins, such as collagen type I, fibronectin, and ICAM-1 [65]. Accumulating evidence indicates TGFβ promotes pancreatic fibrosis through not only increasing ECM components but also down-regulating MMP activity, such as MMP2 [68], MMP3 and MMP9 [69]. As previously discussed, TGFβ can regulate ROS level while NOX-derived ROS associates with fibrosis in pancreas [70]. ROS mediates PSC activation via AP-1 and MAPK signalling, and the fibrotic process by activating AKT and NF-ĸB signalling pathways, up-regulating MMP-9 and Twist, and producing α-SMA and collagen I and III [70, 71]. Increased ROS level links to TGFβ activation and production, suggesting the interplay in the fibrosis process.

Interestingly, evidence also shows that desmoplasia can restrain pancreatic tumorigenesis through regulating immunosuppression. Depletion of fibrosis enhances immunosuppression and undifferentiated tumour cell population, which correlates with PDAC progression and lower survival rate. α-SMA^+^ myofibroblast deletion in PDAC reduces fibrosis development in *Ptf1a*^*cre/+*^;*LSL-Kras*^*G12D/+;*^*Tgfbr2*^*flox/flox*^ transgenic mice [72]. Such myofibroblast-deleted tumours enhanced tumour hypoxia, EMT program and CD44^+^CD133^+^ CSC phenotype, while decreased angiogenesis and cytotoxic CD8^+^/Treg ratio [72]. Sonic hedgehog-deficient tumours also display stromal loss in *Pdx1-Cre;Kras*^*LSL−G12D/+*^;*p53*^*fl/+*^;*Rosa26*^*LSL − YFP/+*^ transgenic mice, with increasing undifferentiated histology, vascularity, EMT gene expression and heightened proliferation [73]. A recent study demonstrates that fibroblast-specific deletion of collagen I, in *FSF-Kras*^*G12D/+;*^*Trp53*^*frt/frt*^;*Pdx1-Flp* transgenic mice, leads to *Cxcl5* upregulation through SOX9, which in turn recruits CD206^+^ARG1^+^ myeloid-derived suppressor cells (MDSCs) and suppresses CD8^+^ T cells [74].

### Impact of TGFβ and ROS in immune evasion

Extensive infiltration by immunosuppressive cell populations promotes PDAC progression. The immunosuppressive cell population includes myeloid-derived suppressor cells (MDSCs), regulatory T cells (Tregs), and tumour-associated macrophages (TAMs). It is clear that TGFβ in tumour microenvironment can regulate differentiation and expansion of MDSCs and Tregs (reviewed in [75]). In the presence of TGFβ, MDSCs show more efficiency in supressing T cell proliferation and inducing Tregs [76]. ROS also influence and are released by Tregs and MDSCs for immune response control. MDSCs can exert immunosuppression through ROS production. The oxidative stress in tumour microenvironment maintains MDSC phenotypes while inhibition of ROS abrogate MDSC suppression on T cells [77]. In line with it, an independent study demonstrates that MDSCs from tumour-bearing mice compromise TGFβ-induced Foxp3^+^CD4^+^CD25^+^ Treg differentiation in a ROS-dependent manner [78]. Tregs is another key immunosuppressive cell population increasing in cancer patients. Such increased Treg prevalence has been demonstrated to be a prognostic factor for PDAC [79–81]. Tumour-derived TGFβ induces Foxp3^+^ Tregs conversion from its CD4^+^CD25^−^ precursors [82, 83]. TGFβ triggers ROS production through activating NOX4 in Tregs. Also, Tregs activation correlates with ROS level [84].

## Targeting ROS and TGFβ for PDAC therapy

**Fig. 3:**
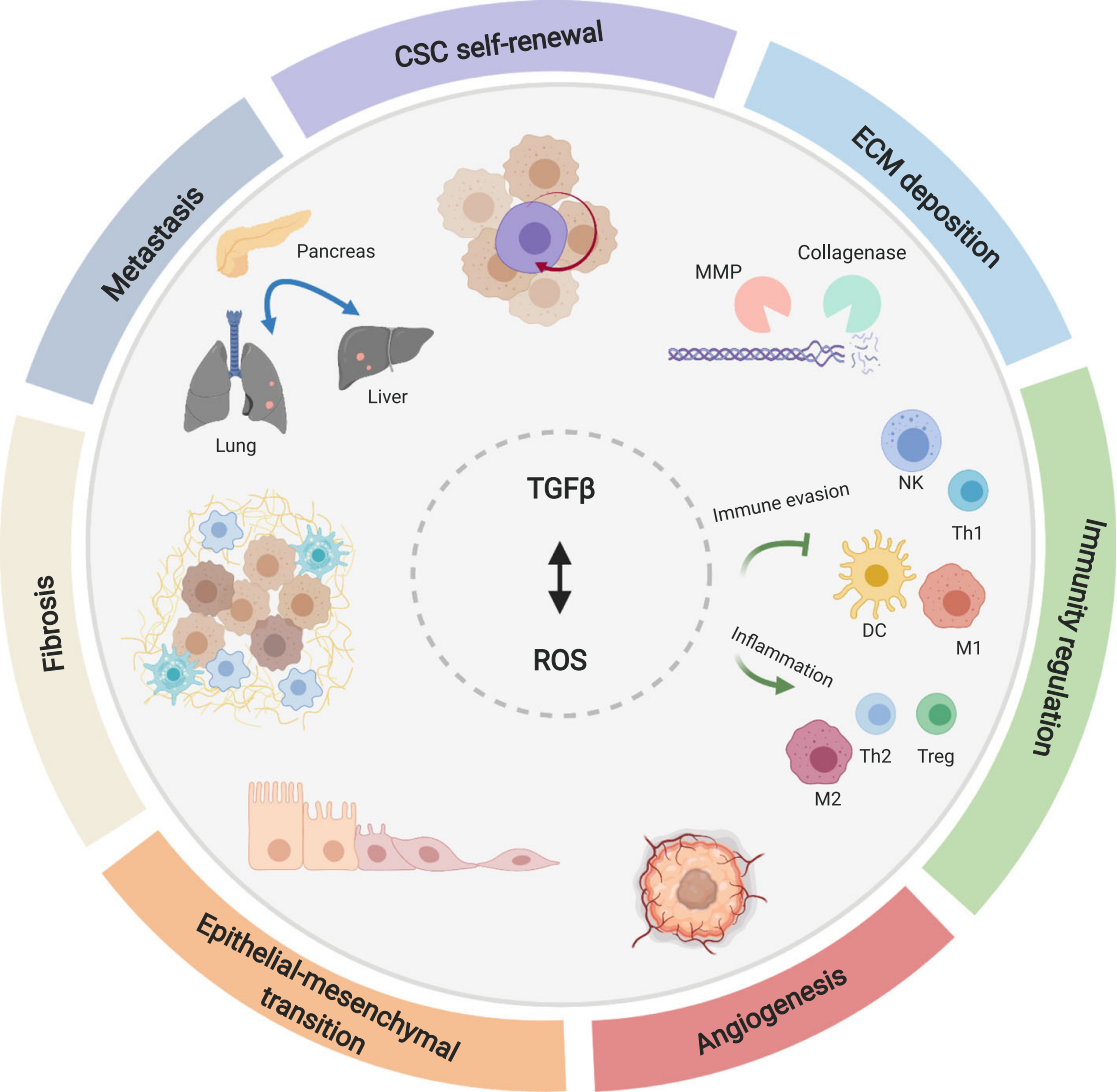
Combined targeting of ROS and TGFβ as cancer therapy. Canonical and non-canonical TGFβ signalling inhibitors could be combined with ROS induction to increase the effectiveness of therapeutic strategies for cancer. These mechanisms would impact fibrosis, immunity regulation, extracellular matrix (ECM) deposition, as well as cancer stem cell (CSC) self-renewal and epithelial-mesenchymal transition, tumour metastasis and angiogenesis. Combinational targeting of TGFβ activators, instead of targeting TGFβ alone, may further increase efficiency and specificity of treatment. Positive regulations (arrows) and negative regulations (inhibitors) are shown in the figure. MMP: matrix metalloproteinases; DC: dendritic cell; NK: natural killer cell; M1: classically-activated macrophage; M2: wound-healing macrophage (also known as alternatively-activated macrophage; Th1: type 1 T helper cell; Th2: type 2 T helper cell; Treg: and regulatory T cells

The ROS levels are higher in cancer cells than in normal cells and hence the cancer cells could be more sensitive than normal cells to the accumulation of ROS, thereby opening a therapeutic opportunity [85]. Two therapeutic strategies of targeting ROS have shown some promise. One is to increase ROS to levels that is toxic for PDAC cells by targeting the enhanced antioxidant mechanisms could kill cancer cells without affecting normal cells [86].The opposite strategy is to restrict ROS production and maintain them at levels where they do not facilitate tumorigenesis [3]. However, since diminished or elevated ROS support different stages of PDAC, the use of antioxidants or ROS regulators for cancer treatment underlines the importance of relative threshold levels of ROS and the stage of tumorigenesis. Moreover, intra-tumour heterogeneity complicates treatments since chemotherapies and radiotherapy can lead to ROS levels that are toxic for most cancer cells but also could support EMT and the metastasis of CSCs. Elevated ROS could then form a feed-forward loop with TGFβ signalling that drives a CSC-supportive niche with ECM and desmoplastic stroma. For all these reasons, the non-canonical pathways of TGFβ are particularly important as therapeutic targets as part of a combined treatment strategy. For instance, JNK is upregulated in CSCs and contributes to their maintenance while promoting chemoresistance of CSCs through prevention of 5-fluorouracil and gemcitabine-induced intracellular ROS production. Therefore, JNK inhibition combined with 5-fluorouracil and/or gemcitabine-based regimens may help to eliminate CSCs [87]. The low level of ROS in CSCs and the active ROS detoxifying systems, elevating the concentration of ROS has also the ability to eliminate CSCs if it is combined with EMT inhibition and other cancer hallmark targeting strategies through TGFβ signalling pathway inhibitors and HIF inhibitors (Fig. 3). Indeed, various chemotherapeutic agents (vinca alkaloids, taxanes, platinum coordination complexes) that can increase ROS levels could be of use in combination therapy to cause cell death while preventing CSC metastatic characteristics. Paclitaxel, the mitotic inhibitory drug that stabilizes microtubules, can indirectly increase ROS generation through Rac1 translocation that in turn induces NOX activity. The anti-inflammatory drug sulfasalazine that has an xc − cystine transporter inhibitory activity, markedly reduces the cystine uptake, GSH level, and growth and viability of human pancreatic cancer cells [88]. Targeting the TGFβ pathway in combination with cysteine depletion by cyst(e)inase could more efficiently avoid CSC metastatic dissemination while inducing pancreatic tumour ferroptosis, a form of cell death that results from the catastrophic accumulation of lipid ROS [89]. If low specificity and toxicity could be overcome for TGFβ signalling, then it could be an attractive target for pharmacological intervention in PDAC.

## Concluding remarks and future perspectives

TGFβ and ROS crosstalk plays an important role throughout pancreatic cancer development. ROS-mediated processes converge with the spatial and temporal activation of TGFβ signalling and thereby differentially affect early tumour growth versus metastatic dissemination. The levels of ROS impact its anti- and pro-tumorigenic effects which in turn depend on cell contexts and the cancer stage. Therefore, these parameters will have an impact on the success of using ROS as a therapeutic target. Due to the intersection of TGFβ signalling pathways with the signalling mediators of ROS, combined targeting of the different signalling branches of TGFβ and ROS pathways could yield improved efficiencies. This combined strategy would aim to target several hallmarks of PDAC that involve specifically eliminating CSCs as well as non-CSCs, and in parallel targeting the tumour microenvironment.

## Data Availability

Not applicable.

## Abbreviations

ADM: acinar-to-ductal metaplasia
AKT: protein kinase B
AMPK: AMP-activated protein kinase
AP-1: activator protein 1
ARNT: aryl hydrocarbon receptor nuclear translocator
ATP: adenosine triphosphate
CDK2: cyclin-dependent kinase 2
c-Jun: also known as activator protein 1
CSC: cancer stem cell
CTGF: connective tissue growth factor
DC: dendritic cell
ECM: extracellular matrix
EGF: epidermal growth factor
EGFR: epidermal growth factor receptor
EMT: epithelial to mesenchymal transition
FAK: focal adhesion kinase
ERK: extracellular signal-regulated kinase
Fra-1: Fos Fosrelated antigen 1
GCL: glutamate cysteine ligase
GS: GSH synthase
GSH: glutathione
HIFs: hypoxia inducible factors
JNK: c-Jun N-terminal kinase
KRAS: Kirsten rat sarcoma viral
LAP: latency-associated peptide
M1: classically-activated macrophage
M2: wound-healing macrophage (also known as alternatively-activated macrophage)
MAPK: mitogen-activated protein kinase
MDSC: myeloid-derived suppressor cell
MEK: mitogen-activated protein kinase kinase
MMP: matrix metalloproteinases
mTOR: mechanistic target of rapamycin
NAC: N-acetylcysteine
NFκB: nuclear factor-κB
NK: natural killer cell
NOXs: NADPH oxidases
PanIN: pancreatic intraepithelial neoplasia
PDAC: pancreatic ductal adenocarcinoma
PSC: pancreatic stellate cell
PI3K: phosphatidylinositol-3 kinase
PKD1: protein kinase D1
PTEN: phosphatase and tensin homolog
Rac1: Rac Family Small GTPase 1
RhoA: Ras homolog family member A
ROCK: Rho-associated protein kinase
ROS: reactive oxygen species
RREB1: Ras responsive element binding protein 1
SARA: smad anchor for receptor activation
TAMs: tumour-associated macrophages
TAK1: TGFβ activated kinase 1
TGFβ: Transforming growth factor β
TGFβR: transforming growth factor β receptor 1/2
Th1: type 1 T helper cell
Th2: type 2 T helper cell
TIGAR: TP53 induced glycolysis regulatory phosphatase
TIMP3: TIMP Metallopeptidase Inhibitor 3
Tp53INP1: tumour protein P53 inducible nuclear protein 1
TRAFs: TNF receptor associated factors
Treg: regulatory T cells
TSP1: thrombospondin-1
VEGF: vascular endothelial growth factor
α-SMA: smooth muscle Actin
